# 2-(4-Isobutyl­phen­yl)-*N*′-[(3*Z*)-2-oxoindolin-3-yl­idene]propano­hydrazide

**DOI:** 10.1107/S160053681201269X

**Published:** 2012-03-28

**Authors:** Shaaban K. Mohamed, Mehmet Akkurt, Mustafa R. Albayati, Kuldip Singh, Herman Potgieter

**Affiliations:** a Chemistry and Environmental Division, Manchester Metropolitan University, Manchester M1 5GD, England; b Department of Physics, Faculty of Sciences, Erciyes University, 38039 Kayseri, Turkey; c Department of Chemistry, University of Leicester, Leicester, England; d School of Research, Enterprise & Innovation, Manchester Metropolitan University, Manchester M1 5GD, England

## Abstract

In the title compound, C_21_H_23_N_3_O_2_, the indolin-2-one group is essentially planar, with a maximum deviation of 0.016 (2) Å for the N atom, and makes a dihedral angle of 84.38 (14)° with the benzene ring. The =N—N(H)—C(=O)—C– torsion angle is 0.9 (3)°. In the crystal, mol­ecules are linked into a three-dimensional network *via* N—H⋯O and C—H⋯O hydrogen bonds. In addition, a C—H⋯π inter­action was observed.

## Related literature

For the pharmaceutical applications of hydrazones, see: Bedia *et al.* (2006 ▶); Rollas *et al.* (2002 ▶). For the pharmaceutical applications of ibuprofen, see: Palaska *et al.* (2002 ▶). For the synthesis of hydrazones, see: Rollas & Küçükgüzel (2007 ▶). For some of our studies on the synthesis of biologically active compounds, see: Mohamed *et al.* (2012*a* ▶,*b* ▶); Soliman *et al.* (2012 ▶). For bond-length data, see: Allen *et al.* (1987 ▶).
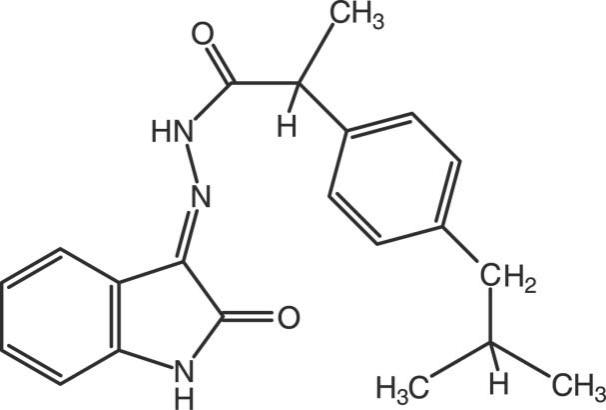


## Experimental

### 

#### Crystal data

C_21_H_23_N_3_O_2_
*M* *_r_* = 349.42
Monoclinic, 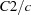

*a* = 30.366 (14) Å
*b* = 7.383 (3) Å
*c* = 21.904 (10) Å
β = 130.311 (8)°
*V* = 3745 (3) Å^3^
*Z* = 8
Mo *K*α radiation
μ = 0.08 mm^−1^
*T* = 150 K
0.35 × 0.21 × 0.10 mm

#### Data collection

Bruker APEX 2000 CCD area-detector diffractometer
Absorption correction: multi-scan (*SADABS*; Sheldrick, 1996 ▶) *T*_min_ = 0.972, *T*_max_ = 0.992
15933 measured reflections
4494 independent reflections
2050 reflections with *I* > 2σ(*I*)
*R*_int_ = 0.096

#### Refinement

*R*[*F* ^2^ > 2σ(*F* ^2^)] = 0.067
*wR*(*F* ^2^) = 0.182
*S* = 0.89
4494 reflections
223 parameters
H-atom parameters constrained
Δρ_max_ = 0.60 e Å^−3^
Δρ_min_ = −0.39 e Å^−3^

### 

Data collection: *APEX2* (Bruker, 2005 ▶); cell refinement: *SAINT* (Bruker, 2005 ▶); data reduction: *SAINT*; program(s) used to solve structure: *SIR97* (Altomare *et al.*, 1999 ▶); program(s) used to refine structure: *SHELXL97* (Sheldrick, 2008 ▶); molecular graphics: *ORTEP-3 for Windows* (Farrugia, 1997 ▶) and *PLATON* (Spek, 2009 ▶); software used to prepare material for publication: *WinGX* (Farrugia, 1999 ▶) and *PLATON*.

## Supplementary Material

Crystal structure: contains datablock(s) global, I. DOI: 10.1107/S160053681201269X/hg5199sup1.cif

Structure factors: contains datablock(s) I. DOI: 10.1107/S160053681201269X/hg5199Isup2.hkl

Supplementary material file. DOI: 10.1107/S160053681201269X/hg5199Isup3.cml

Additional supplementary materials:  crystallographic information; 3D view; checkCIF report

## Figures and Tables

**Table 1:** Hydrogen-bond geometry (Å, °) *Cg*1 is the centroid of the N1/C1/C6–C8 ring.

*D*—H⋯*A*	*D*—H	H⋯*A*	*D*⋯*A*	*D*—H⋯*A*
N1—H1⋯O1^i^	0.86	1.91	2.740 (4)	163
N3—H3*A*⋯O2^ii^	0.86	2.16	2.965 (4)	155
C5—H5⋯O2^ii^	0.93	2.30	3.218 (3)	172
C11—H11*B*⋯*Cg*1^iii^	0.96	2.77	3.703 (4)	164

Symmetry codes: (i) 
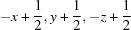
; (ii) 
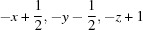
; (iii) 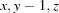.

**Fig. 1.**
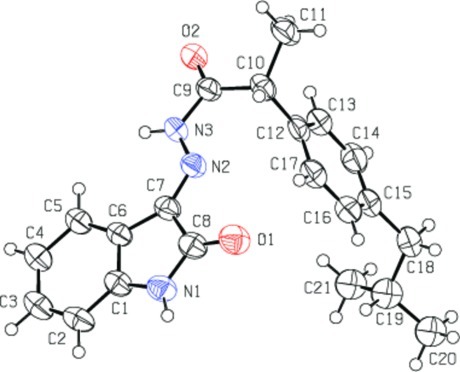
The title compound with the atom numbering scheme. Displacement ellipsoids for non-H atoms are drawn at the 50% probability level.

**Fig. 2.**
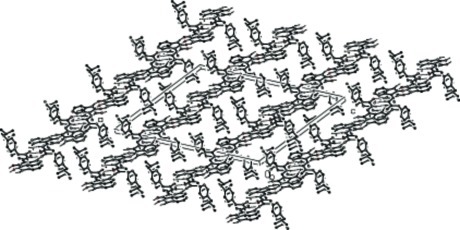
View of the crystal packing and hydrogen bonding of (I) down the b axis. H atoms not involved in hydrogen bonds have been omitted for clarity.

## supplementary crystallographic information

### Comment

Hydrazide-hydrazone compounds are found to be associated with various biological activities such as antimicrobial, anticonvulsant, analgesic, anti-inflammatory, antiplatelet, antitubercular and antitumor properties (Bedia *et al.*, 2006; Rollas *et al.*, 2002; Palaska *et al.*, 2002; Rollas & Küçükgüzel, 2007). Further to our strategy on synthesis of biologically active compounds (Mohamed *et al.*, 2012*a*,*b*; Soliman *et al.* 2012) we get interested to study the functionalization of ibuprofen moiety with the aim of synthesis of potential biologically active compounds based on the core structure of ibuprofen. The title compound (I) was synthesized on condensation of the corresponding hydrazidic acid of ibuprofen with isatin under microwave irradiation and free solvent conditions.

In the title molecule, (Fig. 1), the 1,3-dihydro-2*H*-indol-2-one group (O1/N1/C1—C8) which is essentially planar with a maximum deviation of -0.016 (2) Å for N1 atom, makes a dihedral angle of 84.38 (14)° with the benzene ring (C12—C17). All bond lengths and angles are within normal ranges (Allen *et al.*, 1987). The torsion angles N2—N3—C9—C10, O2—C9—C10—C11, C15—C18—C19—C20 and C15—C18—C19—C21 are 0.9 (3), 21.8 (3), -173.2 (3) and 63.4 (4) °, respectively.

In the crystal structure, the molecules are linked by N—H···O and C—H···O intermolecular hydrogen bonds, forming a three dimensional network (Table 1 and Fig. 2). Furthermore, C—H···π interactions also play an important role in stabilizing the structure (Table 1).

### Experimental

A mixture of an equimolar ratio of 2-(4-isobutylphenyl)propanehydrazide (220 mg) and 1*H*-indole-2,3-dione (147 mg) was ground in a mortar and well mixed with few drops of acetic acid as catalytic agent. The mixture powder has been transferred in a dry conical flask then irradiated under 600 w microwave for 2–3 minutes with intervals every 30 s. The yelow solid product was collected and crystallized from ethanol to afford plate bright yellow crystals in 96% yield with m.p. at 439 - 441 K. A suitable crystals for X-ray diffraction was prepared by slow evaporation of an ethanolic solution of product over two days at room temperature.

### Refinement

All hydrogen atom were located geometrically and refined using a riding model with N—H = 0.86 Å, C—H = 0.93–0.98 Å, and with *U*_iso_ =1.2–1.5*U*_eq_(C,*N*).

### Crystal data

**Table:**

C_21_H_23_N_3_O_2_	*F*(000) = 1488
*M**_r_* = 349.42	*D*_x_ = 1.240 Mg m^−^^3^
Monoclinic, *C*2/*c*	Mo *K*α radiation, λ = 0.71073 Å
Hall symbol: -C 2yc	Cell parameters from 654 reflections
*a* = 30.366 (14) Å	θ = 2.9–23.4°
*b* = 7.383 (3) Å	µ = 0.08 mm^−^^1^
*c* = 21.904 (10) Å	*T* = 150 K
β = 130.311 (8)°	Plate, yellow
*V* = 3745 (3) Å^3^	0.35 × 0.21 × 0.10 mm
*Z* = 8	

### Data collection

**Table:**

Bruker APEX 2000 CCD area-detector diffractometer	4494 independent reflections
Radiation source: fine-focus sealed tube	2050 reflections with *I* > 2σ(*I*)
Graphite monochromator	*R*_int_ = 0.096
phi and ω scans	θ_max_ = 28.7°, θ_min_ = 1.8°
Absorption correction: multi-scan (*SADABS*; Sheldrick, 1996)	*h* = −40→39
*T*_min_ = 0.972, *T*_max_ = 0.992	*k* = −9→9
15933 measured reflections	*l* = −29→28

### Refinement

**Table:**

Refinement on *F*^2^	Primary atom site location: structure-invariant direct methods
Least-squares matrix: full	Secondary atom site location: difference Fourier map
*R*[*F*^2^ > 2σ(*F*^2^)] = 0.067	Hydrogen site location: inferred from neighbouring sites
*wR*(*F*^2^) = 0.182	H-atom parameters constrained
*S* = 0.89	*w* = 1/[σ^2^(*F*_o_^2^) + (0.0771*P*)^2^] where *P* = (*F*_o_^2^ + 2*F*_c_^2^)/3
4494 reflections	(Δ/σ)_max_ < 0.001
223 parameters	Δρ_max_ = 0.60 e Å^−^^3^
0 restraints	Δρ_min_ = −0.39 e Å^−^^3^

### Special details

**Table:**

Geometry. Bond distances, angles *etc*. have been calculated using the rounded fractional coordinates. All su's are estimated from the variances of the (full) variance-covariance matrix. The cell e.s.d.'s are taken into account in the estimation of distances, angles and torsion angles
Refinement. Refinement on *F*^2^ for ALL reflections except those flagged by the user for potential systematic errors. Weighted *R*-factors *wR* and all goodnesses of fit *S* are based on *F*^2^, conventional *R*-factors *R* are based on *F*, with *F* set to zero for negative *F*^2^. The observed criterion of *F*^2^ > σ(*F*^2^) is used only for calculating -*R*-factor-obs *etc*. and is not relevant to the choice of reflections for refinement. *R*-factors based on *F*^2^ are statistically about twice as large as those based on *F*, and *R*-factors based on ALL data will be even larger.

### Fractional atomic coordinates and isotropic or equivalent isotropic displacement parameters (Å^2^)

**Table:**

	*x*	*y*	*z*	*U*_iso_*/*U*_eq_	
O1	0.27378 (8)	0.1979 (3)	0.28135 (11)	0.0610 (8)	
O2	0.30262 (7)	−0.3536 (2)	0.51498 (11)	0.0551 (7)	
N1	0.21763 (9)	0.3981 (3)	0.28414 (12)	0.0513 (8)	
N2	0.26682 (8)	−0.0134 (3)	0.38256 (12)	0.0441 (7)	
N3	0.26590 (8)	−0.1154 (3)	0.43261 (12)	0.0438 (7)	
C1	0.19248 (10)	0.4090 (3)	0.31931 (14)	0.0449 (9)	
C2	0.16039 (11)	0.5488 (4)	0.31409 (15)	0.0536 (9)	
C3	0.13834 (11)	0.5291 (4)	0.35241 (16)	0.0552 (10)	
C4	0.14805 (11)	0.3748 (4)	0.39497 (16)	0.0518 (10)	
C5	0.18120 (10)	0.2344 (3)	0.40110 (14)	0.0462 (8)	
C6	0.20363 (9)	0.2513 (3)	0.36274 (13)	0.0406 (8)	
C7	0.23936 (10)	0.1382 (3)	0.35482 (14)	0.0426 (8)	
C8	0.24645 (11)	0.2419 (4)	0.30246 (15)	0.0480 (9)	
C9	0.30217 (10)	−0.2603 (3)	0.46825 (15)	0.0445 (8)	
C10	0.34176 (10)	−0.2953 (3)	0.45024 (15)	0.0469 (9)	
C11	0.36252 (12)	−0.4915 (4)	0.47037 (18)	0.0631 (11)	
C12	0.39149 (10)	−0.1604 (3)	0.49514 (16)	0.0445 (9)	
C13	0.43620 (11)	−0.1733 (4)	0.57647 (16)	0.0511 (10)	
C14	0.48254 (11)	−0.0548 (4)	0.61611 (17)	0.0571 (10)	
C15	0.48610 (12)	0.0796 (4)	0.57581 (19)	0.0556 (10)	
C16	0.44102 (12)	0.0942 (4)	0.49473 (19)	0.0583 (11)	
C17	0.39438 (12)	−0.0220 (4)	0.45516 (17)	0.0533 (10)	
C18	0.53791 (12)	0.2047 (4)	0.6173 (2)	0.0705 (13)	
C19	0.52905 (13)	0.3956 (4)	0.63358 (19)	0.0679 (7)	
C20	0.58104 (12)	0.5126 (4)	0.66581 (18)	0.0679 (7)	
C21	0.51718 (12)	0.3939 (4)	0.68999 (18)	0.0679 (7)	
H1	0.21500	0.48110	0.25440	0.0620*	
H2	0.15370	0.65350	0.28560	0.0640*	
H3	0.11640	0.62220	0.34950	0.0660*	
H3A	0.24300	−0.08990	0.44180	0.0530*	
H4	0.13230	0.36450	0.41980	0.0620*	
H5	0.18820	0.13090	0.43040	0.0550*	
H10	0.31960	−0.27740	0.39270	0.0560*	
H11A	0.38740	−0.51360	0.45840	0.0950*	
H11B	0.32980	−0.57130	0.43910	0.0950*	
H11C	0.38340	−0.51290	0.52630	0.0950*	
H13	0.43530	−0.26330	0.60530	0.0610*	
H14	0.51190	−0.06620	0.67120	0.0690*	
H16	0.44200	0.18430	0.46600	0.0700*	
H17	0.36430	−0.00710	0.40050	0.0640*	
H18A	0.54900	0.21270	0.58470	0.0850*	
H18B	0.56990	0.15020	0.66790	0.0850*	
H19	0.49540	0.44780	0.58270	0.0810*	
H20A	0.58750	0.51340	0.62830	0.1020*	
H20B	0.61440	0.46460	0.71610	0.1020*	
H20C	0.57410	0.63400	0.67350	0.1020*	
H21A	0.48390	0.32030	0.66820	0.1020*	
H21B	0.51010	0.51530	0.69750	0.1020*	
H21C	0.54990	0.34500	0.74060	0.1020*	

### Atomic displacement parameters (Å^2^)

**Table:**

	*U*^11^	*U*^22^	*U*^33^	*U*^12^	*U*^13^	*U*^23^
O1	0.0792 (14)	0.0562 (13)	0.0671 (13)	0.0042 (10)	0.0561 (12)	−0.0018 (10)
O2	0.0503 (11)	0.0447 (11)	0.0692 (13)	0.0100 (8)	0.0381 (10)	0.0125 (10)
N1	0.0603 (14)	0.0449 (14)	0.0494 (13)	0.0086 (11)	0.0358 (12)	0.0091 (11)
N2	0.0440 (12)	0.0377 (13)	0.0491 (12)	0.0025 (10)	0.0294 (11)	−0.0028 (10)
N3	0.0415 (12)	0.0376 (12)	0.0540 (13)	0.0078 (9)	0.0316 (11)	0.0026 (10)
C1	0.0454 (15)	0.0418 (16)	0.0387 (14)	0.0060 (12)	0.0232 (13)	−0.0001 (12)
C2	0.0568 (16)	0.0387 (16)	0.0482 (16)	0.0117 (13)	0.0263 (14)	0.0041 (13)
C3	0.0562 (17)	0.0466 (17)	0.0532 (16)	0.0140 (13)	0.0311 (15)	−0.0016 (14)
C4	0.0547 (16)	0.0478 (17)	0.0545 (17)	0.0088 (13)	0.0360 (14)	−0.0005 (14)
C5	0.0448 (14)	0.0406 (15)	0.0456 (14)	0.0058 (12)	0.0258 (13)	−0.0013 (12)
C6	0.0394 (13)	0.0342 (14)	0.0396 (13)	0.0044 (11)	0.0217 (12)	−0.0016 (11)
C7	0.0417 (14)	0.0358 (14)	0.0417 (14)	0.0020 (11)	0.0231 (12)	−0.0029 (11)
C8	0.0532 (16)	0.0434 (16)	0.0435 (14)	0.0009 (13)	0.0295 (14)	−0.0058 (13)
C9	0.0387 (14)	0.0336 (14)	0.0517 (15)	0.0017 (11)	0.0250 (13)	−0.0039 (13)
C10	0.0461 (15)	0.0420 (16)	0.0503 (15)	0.0079 (12)	0.0302 (13)	−0.0044 (12)
C11	0.0606 (18)	0.0435 (18)	0.081 (2)	0.0089 (14)	0.0439 (17)	−0.0083 (15)
C12	0.0421 (14)	0.0431 (16)	0.0563 (16)	0.0105 (12)	0.0354 (14)	−0.0026 (13)
C13	0.0471 (15)	0.0458 (17)	0.0614 (18)	0.0061 (13)	0.0356 (15)	0.0005 (14)
C14	0.0444 (16)	0.0588 (19)	0.0588 (17)	0.0081 (14)	0.0292 (15)	−0.0030 (15)
C15	0.0484 (16)	0.0472 (18)	0.079 (2)	0.0078 (13)	0.0447 (17)	−0.0033 (15)
C16	0.0610 (18)	0.0468 (18)	0.081 (2)	0.0095 (14)	0.0522 (18)	0.0074 (16)
C17	0.0545 (17)	0.0521 (18)	0.0603 (17)	0.0114 (14)	0.0403 (15)	0.0012 (15)
C18	0.0526 (17)	0.058 (2)	0.104 (3)	0.0077 (15)	0.0520 (19)	−0.0014 (18)
C19	0.0653 (11)	0.0599 (12)	0.0753 (12)	0.0005 (9)	0.0441 (10)	0.0013 (10)
C20	0.0653 (11)	0.0599 (12)	0.0753 (12)	0.0005 (9)	0.0441 (10)	0.0013 (10)
C21	0.0653 (11)	0.0599 (12)	0.0753 (12)	0.0005 (9)	0.0441 (10)	0.0013 (10)

### Geometric parameters (Å, º)

**Table:**

O1—C8	1.227 (5)	C16—C17	1.380 (5)
O2—C9	1.227 (4)	C18—C19	1.520 (5)
N1—C1	1.394 (5)	C19—C21	1.494 (6)
N1—C8	1.343 (4)	C19—C20	1.515 (6)
N2—N3	1.345 (3)	C2—H2	0.9300
N2—C7	1.289 (3)	C3—H3	0.9300
N3—C9	1.362 (4)	C4—H4	0.9300
N1—H1	0.8600	C5—H5	0.9300
N3—H3A	0.8600	C10—H10	0.9800
C1—C6	1.399 (3)	C11—H11A	0.9600
C1—C2	1.373 (5)	C11—H11B	0.9600
C2—C3	1.379 (5)	C11—H11C	0.9600
C3—C4	1.378 (4)	C13—H13	0.9300
C4—C5	1.390 (5)	C14—H14	0.9300
C5—C6	1.388 (5)	C16—H16	0.9300
C6—C7	1.466 (4)	C17—H17	0.9300
C7—C8	1.506 (4)	C18—H18A	0.9700
C9—C10	1.510 (5)	C18—H18B	0.9700
C10—C12	1.523 (4)	C19—H19	0.9800
C10—C11	1.527 (4)	C20—H20A	0.9600
C12—C13	1.377 (4)	C20—H20B	0.9600
C12—C17	1.385 (4)	C20—H20C	0.9600
C13—C14	1.385 (5)	C21—H21A	0.9600
C14—C15	1.378 (5)	C21—H21B	0.9600
C15—C18	1.518 (5)	C21—H21C	0.9600
C15—C16	1.376 (5)		
			
C1—N1—C8	111.6 (2)	C3—C2—H2	121.00
N3—N2—C7	121.5 (3)	C2—C3—H3	119.00
N2—N3—C9	118.1 (3)	C4—C3—H3	119.00
C8—N1—H1	124.00	C3—C4—H4	120.00
C1—N1—H1	124.00	C5—C4—H4	120.00
C9—N3—H3A	121.00	C4—C5—H5	121.00
N2—N3—H3A	121.00	C6—C5—H5	121.00
N1—C1—C2	127.6 (2)	C9—C10—H10	108.00
C2—C1—C6	121.9 (3)	C11—C10—H10	108.00
N1—C1—C6	110.5 (2)	C12—C10—H10	108.00
C1—C2—C3	117.9 (3)	C10—C11—H11A	109.00
C2—C3—C4	121.6 (3)	C10—C11—H11B	109.00
C3—C4—C5	120.5 (3)	C10—C11—H11C	109.00
C4—C5—C6	118.9 (2)	H11A—C11—H11B	110.00
C1—C6—C7	105.4 (3)	H11A—C11—H11C	109.00
C5—C6—C7	135.3 (2)	H11B—C11—H11C	109.00
C1—C6—C5	119.3 (3)	C12—C13—H13	119.00
N2—C7—C8	115.7 (3)	C14—C13—H13	119.00
C6—C7—C8	106.4 (2)	C13—C14—H14	119.00
N2—C7—C6	137.9 (3)	C15—C14—H14	119.00
O1—C8—N1	125.7 (3)	C15—C16—H16	119.00
O1—C8—C7	128.1 (3)	C17—C16—H16	119.00
N1—C8—C7	106.1 (3)	C12—C17—H17	119.00
O2—C9—N3	119.2 (3)	C16—C17—H17	119.00
N3—C9—C10	118.0 (2)	C15—C18—H18A	108.00
O2—C9—C10	122.7 (2)	C15—C18—H18B	108.00
C9—C10—C11	109.7 (3)	C19—C18—H18A	108.00
C9—C10—C12	110.1 (2)	C19—C18—H18B	108.00
C11—C10—C12	112.5 (3)	H18A—C18—H18B	107.00
C13—C12—C17	117.2 (3)	C18—C19—H19	108.00
C10—C12—C13	121.8 (3)	C20—C19—H19	108.00
C10—C12—C17	121.1 (2)	C21—C19—H19	108.00
C12—C13—C14	121.3 (3)	C19—C20—H20A	109.00
C13—C14—C15	121.5 (3)	C19—C20—H20B	109.00
C14—C15—C16	117.3 (3)	C19—C20—H20C	109.00
C14—C15—C18	122.4 (3)	H20A—C20—H20B	110.00
C16—C15—C18	120.4 (3)	H20A—C20—H20C	110.00
C15—C16—C17	121.4 (3)	H20B—C20—H20C	109.00
C12—C17—C16	121.4 (3)	C19—C21—H21A	109.00
C15—C18—C19	115.6 (4)	C19—C21—H21B	110.00
C18—C19—C21	111.1 (3)	C19—C21—H21C	110.00
C20—C19—C21	110.8 (3)	H21A—C21—H21B	109.00
C18—C19—C20	110.4 (4)	H21A—C21—H21C	109.00
C1—C2—H2	121.00	H21B—C21—H21C	109.00
			
C1—N1—C8—C7	0.3 (3)	C6—C7—C8—O1	179.0 (3)
C8—N1—C1—C2	179.5 (3)	C6—C7—C8—N1	0.5 (3)
C8—N1—C1—C6	−1.1 (3)	N2—C7—C8—O1	2.1 (4)
C1—N1—C8—O1	−178.2 (3)	O2—C9—C10—C11	21.8 (3)
N3—N2—C7—C8	177.3 (2)	O2—C9—C10—C12	−102.5 (3)
C7—N2—N3—C9	−171.0 (2)	N3—C9—C10—C11	−160.3 (2)
N3—N2—C7—C6	1.7 (5)	N3—C9—C10—C12	75.4 (3)
N2—N3—C9—C10	0.9 (3)	C9—C10—C12—C13	73.9 (4)
N2—N3—C9—O2	179.0 (2)	C9—C10—C12—C17	−108.0 (3)
N1—C1—C6—C7	1.4 (3)	C11—C10—C12—C13	−48.8 (4)
C2—C1—C6—C5	0.7 (4)	C11—C10—C12—C17	129.4 (3)
C2—C1—C6—C7	−179.2 (2)	C10—C12—C13—C14	177.0 (3)
N1—C1—C6—C5	−178.8 (2)	C17—C12—C13—C14	−1.2 (5)
C6—C1—C2—C3	−0.9 (4)	C10—C12—C17—C16	−176.1 (3)
N1—C1—C2—C3	178.5 (3)	C13—C12—C17—C16	2.1 (5)
C1—C2—C3—C4	0.2 (4)	C12—C13—C14—C15	−0.6 (6)
C2—C3—C4—C5	0.7 (5)	C13—C14—C15—C16	1.5 (6)
C3—C4—C5—C6	−0.9 (4)	C13—C14—C15—C18	−176.8 (4)
C4—C5—C6—C7	−180.0 (3)	C14—C15—C16—C17	−0.6 (6)
C4—C5—C6—C1	0.2 (4)	C18—C15—C16—C17	177.7 (4)
C1—C6—C7—C8	−1.2 (3)	C14—C15—C18—C19	−104.2 (4)
C5—C6—C7—C8	179.1 (3)	C16—C15—C18—C19	77.6 (5)
C5—C6—C7—N2	−5.1 (6)	C15—C16—C17—C12	−1.2 (6)
C1—C6—C7—N2	174.7 (3)	C15—C18—C19—C20	−173.2 (3)
N2—C7—C8—N1	−176.4 (2)	C15—C18—C19—C21	63.4 (4)

### Hydrogen-bond geometry (Å, º)

Cg1 is the centroid of the N1/C1/C6–C8 ring.

**Table:**

*D*—H···*A*	*D*—H	H···*A*	*D*···*A*	*D*—H···*A*
N1—H1···O1^i^	0.86	1.91	2.740 (4)	163
N3—H3*A*···O2^ii^	0.86	2.16	2.965 (4)	155
C5—H5···O2^ii^	0.93	2.30	3.218 (3)	172
C11—H11*B*···*Cg*1^iii^	0.96	2.77	3.703 (4)	164

Symmetry codes: (i) −*x*+1/2, *y*+1/2, −*z*+1/2; (ii) −*x*+1/2, −*y*−1/2, −*z*+1; (iii) *x*, *y*−1, *z*.
